# Functional Recovery of Contused Spinal Cord in Rat with the Injection of Optimal‐Dosed Cerium Oxide Nanoparticles

**DOI:** 10.1002/advs.201700034

**Published:** 2017-07-08

**Authors:** Jong‐Wan Kim, Chinmaya Mahapatra, Jin‐Young Hong, Min Soo Kim, Kam W. Leong, Hae‐Won Kim, Jung Keun Hyun

**Affiliations:** ^1^ Department of Nanobiomedical Science and BK21 PLUS NBM Global Research Center for Regenerative Medicine Dankook University Cheonan 330‐714 Republic of Korea; ^2^ Institute of Tissue Regeneration Engineering (ITREN) Dankook University Cheonan 330‐714 Republic of Korea; ^3^ Department of Biomedical Engineering Columbia University New York NY 10027 USA; ^4^ Department of Biomaterials Science School of Dentistry Dankook University Cheonan 330‐714 Republic of Korea; ^5^ Department of Rehabilitation Medicine College of Medicine Dankook University Cheonan 330‐714 Republic of Korea

**Keywords:** anti‐inflammation, cerium oxide nanoparticles, functional recovery, reactive oxygen species, spinal cord injury

## Abstract

Spinal cord injury (SCI) produces excess reactive oxygen species (ROS) that can exacerbate secondary injury and lead to permanent functional impairment. Hypothesizing that cerium oxide nanoparticles (CONPs) as an effective ROS scavenger may offset this damaging effect, it is first demonstrated in vitro that CONPs suppressed inducible nitric oxide synthase (iNOS) generation and enhanced cell viability of hydrogen peroxide (H_2_O_2_)‐insulted cortical neurons. Next, CONPs are administered at various does (50–4000 µg mL^−1^) to a contused spinal cord rat model and monitored the disease progression for up to eight weeks. At one day postinjury, the number of iNOS+ cells decreases in the treated groups compared with the control. At one week, the cavity size and inflammatory cells are substantially reduced, and the expression of proinflammatory and apoptotic molecules is downregulated with a concurrent upregulation of anti‐inflammatory cytokine. By eight weeks, the treated groups show significantly improved locomotor functions compared with the control. This study shows for the first time that injection of optimal‐dosed CONPs alone into contusion‐injured spinal cord of rats can reduce ROS level, attenuate inflammation and apoptosis, and consequently help locomotor functional recovery, adding a promising and complementary strategy to the other treatments of acute SCI.

## 1 Introduction

Spinal cord injury (SCI) is one of the most devastating lesions, lacking satisfactory treatment in clinical settings. Compounding the problem is that a typical SCI patient is young with an average age less than 40 years; thus a majority of patients suffer from sensory, motor, and autonomic dysfunctions throughout their lifetime.^1^ Trauma to the spinal cord induces secondary injury, including inflammation, apoptosis, vascular abnormality, glutamate excitotoxicity, free radical formation, and lipid peroxidation, which leads to glial and fibrous scar formation and permanent functional deterioration.^2^ The inflammatory process is considered a key component of secondary injury, detrimental to neuronal regeneration when improperly modulated like the conditions with dominant population of proinflammatory macrophages.^3^

Reactive oxygen species (ROS) dramatically increases during the inflammatory process following spinal cord trauma, contributing to secondary injury and concomitant neuronal cell death, focal axonal degeneration, neuropathic pain, and locomotor dysfunction.^4^ In fact, high levels of ROS are prevalent in various pathological conditions such as aging and death, neurodegenerative disorders, diabetes, arthritis, cancer, and cardiovascular diseases. Therefore, reduction of ROS level would be expected to aid the recovery of biological functions of cells in acute injury conditions. Previous studies revealed that ROS scavengers could attenuate neuronal cell death and apoptosis, and locomotor dysfunctions ascribed to elevated ROS following SCI.[[qv: 4b,5]]

Cerium oxide nanoparticles (CONPs) are nontoxic nanomaterials that have the ability to act as oxidation and reduction catalyst due to the variable oxidation state of cerium (i.e., Ce^3+^/Ce^4+^). Thus they have shown interesting biological properties, including ROS scavenging.^6^ The cellular responses to CONPs vary depending on the cell type. Particularly for neural applications, CONPs increase the survival, proliferation, and differentiation of progenitor cells in vitro due to the protection against endogenous peroxynitrite and Aβ‐induced mitochondrial fragmentation.[[qv: 6c]] Furthermore, the ability of CONPs to cross blood brain barrier was observed in murine model of multiple sclerosis^7^ and ischemia stroke,^8^ suggesting the possible uses in brain disease and injury. CONPs have also shown to be effective in protecting adult cardiac progenitor cells against ROS‐induced stress,^9^ and in reducing inflammation and radiation‐induced damage in internal organs in mice.^10^ On the other hand, some studies have reported controversial effects of CONPs; when applied to human lung epithelial cells, adverse cellular responses including increased ROS, induction of oxidative‐stress genes, and apoptotic cell death were observed.^11^ This is primarily due to the different (possibly higher) doses used and the variable oxidation/reduction state of the CONPs as well as the cell‐dependent responses of nanoparticles such as endocytic activity and toxicity sensitivity. In fact, CONPs, when dosed at high concentrations, are effective in inducing apoptosis of cancerous cells, by increasing the ROS levels, which is associated with different physiological pH levels and the altered catalytic reactions of CONPs.^12^

Based on these series of reports that highlighted the influence of CONPs in controlling the ROS level of cells, we hypothesize that CONPs, if applied at the right dose, may have a therapeutic effect in an acute injury of spinal cord, where a harsh inflammatory condition with substantial generation of ROS is considered a critical barrier to overcome for the repair and regenerative processes. Here we apply various doses of CONPs first to the in vitro hydrogen peroxide (H_2_O_2_) model in cortical neurons, to determine optimal concentrations, and then apply to in vivo SCI model in rat to examine the therapeutic efficacy in reducing the level of ROS and associated molecules, as well as in improving the functional recovery. This is the first report demonstrating the in vivo therapeutic effect of CONPs in acute SCI, thus suggesting a new strategy to the treatments of acute SCI with only nanomaterials.

## 2 Results and Discussion

### 2.1 CONPs Prepared for the Intracellular Delivery

**Figure 1:**
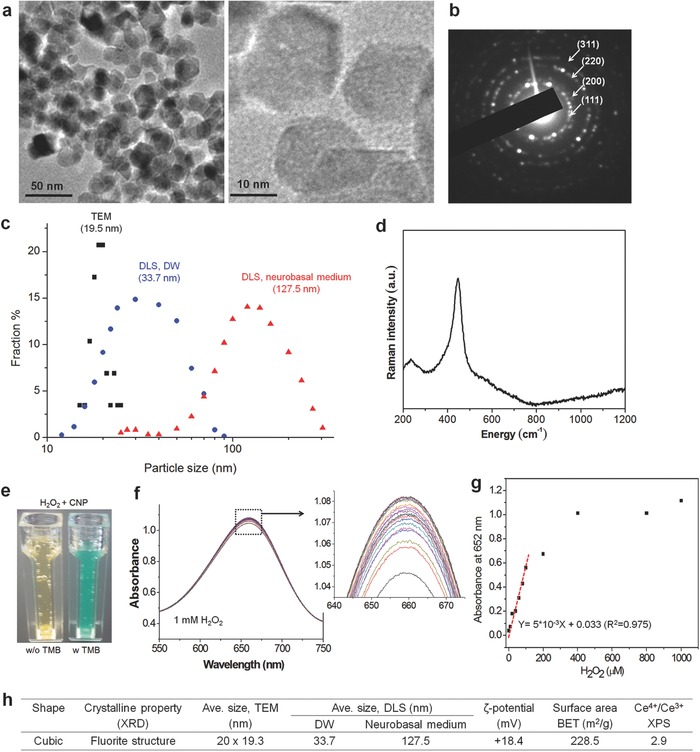
Characteristics of cerium oxide nanoparticles (CONPs). a) TEM images of CONPs at low and high magnification, and b) selected area diffraction pattern of the crystal. c) Size distribution of CONPs, calculated from TEM images and also measured by DLS (in DW or neurobasal medium). d) Raman spectrum. e–g) Oxidase‐like activity of CONPs, evaluated by monitoring the redox reaction between TMB and H_2_O_2_ in the presence of the CONPs; optical view showing a color change by the reaction (e), UV–vis spectroscopic intensity measured time‐dependently (20 cycles per 5 min) at a broad wavelength scan using 1 × 10^−3^ m H_2_O_2_ (f) and then recorded at a specific peak 652 nm with varying H_2_O_2_ dose up to 1000 µm (showing a linear relationship up to ≈100 µm) (g). h) Summary of properties including shape, size (by TEM and DLS method), ζ‐potential, surface area (by BET), and Ce atomic oxidation status (Ce^4+^/Ce^3+^, by XPS).^13^

The typical morphology of the CONPs was observed by transmission electron microscopy (TEM) (**Figure** **1**a,b). Nanoparticles with uniform sizes in an angular form were synthesized reproducibly by a hydrothermal process. Inset image shows the selected area diffraction pattern, revealing a dotted crystal pattern that is typical of cerium oxide. Based on TEM images, the size of CONPs was measured to be 19.5 nm on average. The hydrodynamic size of the nanoparticles, as measured by a dynamic laser scattering (DLS) assay, showed 33.7 and 127.5 nm in distilled water and neurobasal medium, respectively, suggesting the CONPs are considered to disperse mostly in the form of small clusters (a hundred of nanometers) of single nanoparticles within a cell culture medium (Figure 1c). The Raman spectrum of the nanoparticles reveals the pattern typical of cerium oxide (Figure 1d). The oxidase‐like activity of CONPs was also evaluated by monitoring the redox reaction between TMB (3,3′,5,5′‐tetramethyl‐benzidine) and H_2_O_2_ in the presence of the CONPs. An optical view clarified a color change by the reaction (Figure 1e). The UV–vis spectroscopic analysis of the reaction with 1 × 10^−3^ m H_2_O_2_, as measured time‐dependently (20 cycles per 5 min) at a broad wavelength scan, showed a gradual increase of a maximal peak (≈660 nm) with time (Figure 1f). When recorded at a specific peak (652 nm) a linear relationship in intensity was noticed with respect to H_2_O_2_ dose up to ≈100 µm (Figure 1g). The X‐ray photoelectron spectroscopy (XPS) analysis showed the chemical bonding peaks related to Ce^3+^ and Ce^4+^ at the corresponding binding energies (Figure 1h). Other physicochemical properties of the CONPs are also summarized (Figure 1g). The ζ‐potential of the CONPs was moderately positive (+18.4 mV), presumably due to the presence of cations on the surface. Furthermore, the nanoparticles exhibited a high specific surface area of 228 m^2^ g^−1^, attributed to the small size of the nanoparticles. The high surface area also suggests a favorable interaction with the molecules in the biological environment, such as scavenging of ROS through surface reaction and oxidation status change of Ce^4+^/Ce^3+^. The nanoparticles showed a higher incorporation of +4 oxidation status than +3 (with Ce^4+^/Ce^3+^ = 2.9), implying that the CONPs possibly consume ROS effectively at the cost of changing their status primarily from Ce^4+^ to Ce^3+^.^13^

### 2.2 CONPs Internalized into Cortical Neurons In Vitro

**Figure 2:**
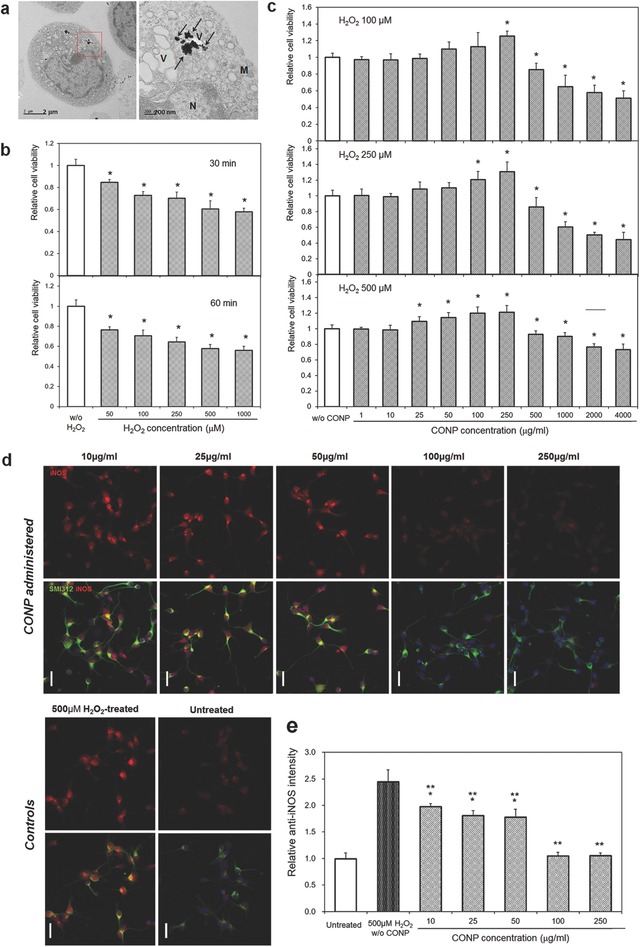
a) CONP (100 µg mL^−1^) internalization to H_2_O_2_ (500 × 10^−6^ m)‐insulted cortical neurons, visualized by TEM; right is a magnified image of a red box in left. Black arrows indicate CONPs (N: nucleus, M: mitochondria, V: vesicle). b–e) Effects of CONP administration to H_2_O_2_‐modeled cortical neurons in vitro: Neuronal viability, after H_2_O_2_ treatment at varying doses of 50 × 10^−6^ m to 1000 × 10^−6^ m for 30 or 60 min, is reduced dose‐dependently (b); *p < 0.05 compared with control by Mann–Whitney U test. Administration of CONP at varying concentrations of 1–4000 µg mL^−1^ to the H_2_O_2_‐insulted neurons recovers the cell viability (c); H_2_O_2_ varied at 100 × 10^−6^ m, 250 × 10^−6^ m, or 500 × 10^−6^ m; *p < 0.05 compared with control by Mann–Whitney U test. In vitro iNOS generation assay (d, e); representative images of anti‐iNOS (red) and anti‐SMI312 (green) positive cortical neurons (scale bar = 20 µm), and the relative intensity of anti‐iNOS levels following 500 × 10^−6^ m H_2_O_2_‐treatment and the concomitant application of various concentrations of CONPs; *p < 0.05 compared with untreated control group, and **p < 0.05 compared with H_2_O_2_‐treated group, by Kruskal–Wallis test with Bonferroni correction.

When we applied CONPs (100 µg mL^−1^) to the primary cultured and H_2_O_2_ (500 × 10^−6^ m)‐insulted cortical neurons of Sprague‐Dawley (SD) rats, CONPs were well internalized into the cytoplasm of neurons, as shown in TEM ultrastructural images (**Figure** **2**a). The CONPs have recently been shown to internalize the cells through the ATP dependent clathrin‐ and caveolae‐mediated endocytic pathways.^14^ The administered CONPs are located in the cytoplasm abundantly, but not in the nucleus of cortical neurons,^14^ suggesting the possible involvement of the nanoparticles in the intracellular regulation of ROS, synthesized in the injured neurons. The cortical neurons appeared to have some vacuoles within cytoplasm, which being considered to result from the H_2_O_2_ treatment (not CONP), as the cytoplasmic vacuolation and cellular swelling were a typical phenomenon observed in cortical neurons when insulted with ROS.^15^

### 2.3 CONPs Administered to H_2_O_2_‐Insulted Cortical Neurons at Proper Concentrations Reduce iNOS Generation and Improve Cell Viability

To investigate the effects of CONPs on the ROS generation in cortical neurons, we in vitro modeled the ROS‐insulting conditions using H_2_O_2_. When various concentrations of H_2_O_2_ (from 50 × 10^−6^ to 1000 × 10^−6^ m) were added to cortical neurons for 30 or 60 min, the neuronal cell viability decreased at all concentrations in a dose‐dependent manner (Figure 2b). Following this we applied three doses (100 × 10^−6^, 250 × 10^−6^, and 500 × 10^−6^ m) of H_2_O_2_ for 30 min to examine the effects of CONPs on the H_2_O_2_‐induced neurons (Figure 2c). At all doses of H_2_O_2_, CONPs applied up to 250 µg mL^−1^ increased the neuronal cell survival, in a concentration‐dependent manner, as examined by the MTT assay, and the maximal MTT cell viability was attained at 250 µg mL^−1^ CONPs, to be ≈120% of H_2_O_2_‐insulted control; however, CONPs added at higher concentrations reduced the MTT cell viability, showing a cytotoxicity (Figure 2c). The live/dead cell assay was also performed to count the fraction of cells alive under the same culture conditions; results demonstrated a trend similar to that observed in the MTT assay (Figure S1, Supporting Information). The cellular toxicity, due to the treatment of high concentrations of CONPs (over 250 µg mL^−1^), appears to be attenuated as the H_2_O_2_ dose increases. Previous studies showed the cellular toxicity of CONPs mainly in a dose‐16 and cell‐dependent manner.^17^ Based on the cell viability results, the CONPs treated up to 250 µg mL^−1^ were considered to be safe for the primary cortical neurons in vitro. Furthermore, the CONPs were effective in enhancing the neuronal viability under in vitro modeled ROS‐insulting conditions; the finding also highlights the importance of identifying the threshold for safe application without inducing cellular toxicity.

We further analyzed the cellular generation of iNOS. The immunocytochemistry of iNOS showed substantial number of cells were positive for iNOS in the 500 × 10^−6^ m H_2_O_2_ group, which however, was greatly reduced in the CONP‐treated groups (Figure 2d). The quantification of fluorescence intensity revealed that the H_2_O_2_ treated neurons generated iNOS significantly (≈2.5 times higher than normal control); however, the administration of CONPs at 10 µg mL^−1^ or more successfully reduced the iNOS level, and in particular, 100 or 250 µg mL^−1^ of CONPs suppressed the generation of iNOS even down to the level of H_2_O_2_‐free control group (Figure 2e). This suppression of iNOS generation in the in vitro oxidative‐stressed neurons, owing to the treatment of CONPs, should lead to the recovery of cells from damage, consequently enhancing the cell viability. The results confirming the effects of CONPs treated at proper concentrations on neurons under in vitro oxidative stress environments entail further investigations into in vivo responses in an animal model.

### 2.4 In Vivo Administration of CONPs to Spinal Cord Injury Reduces Injury Cavity Size, Inflammatory Cell Population, and iNOS Generation

A moderate contusion injury (200 kdyn) to T9 level of adult SD rats was made to generate the SCI in vivo model,^18^ and various concentrations of CONPs were applied to the injury. First, the CONPs were injected once at either of the concentrations (50, 100, 250, 500, 1000, 2000, or 4000 µg mL^−1^), and the animals were sacrificed at one week. Based on the results at this short period, the concentrations of CONPs were then narrowed (250, 500, 1000, and 2000 µg mL^−1^) in the animal study for a longer period of eight weeks. After one week or eight weeks following the treatment, the animals were sacrificed and lesions were examined histologically.

**Figure 3:**
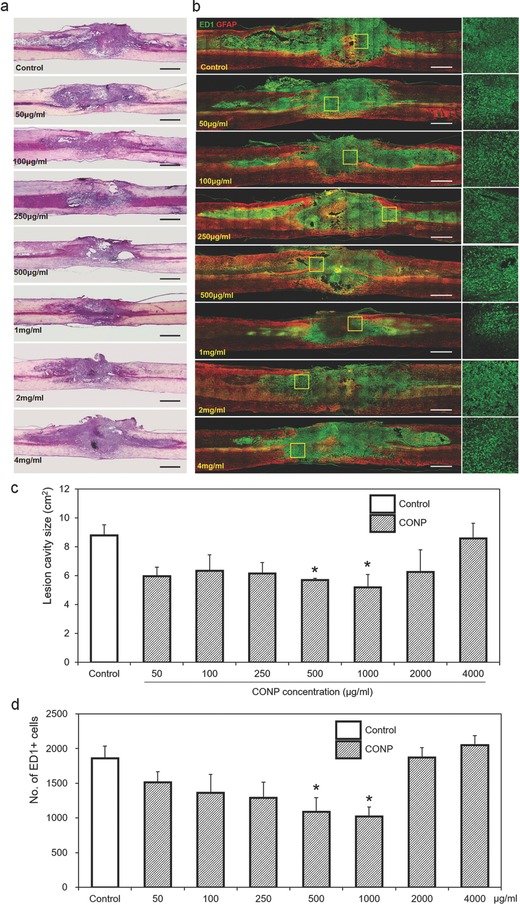
Representative a) H&E and b) immunohistochemical images of the injured spinal cord one week after the administration of CONPs with different concentrations from 50 to 4000 µg mL^−1^. The yellow boxes magnified on right side. c) Size of lesion cavity measured from the sagittal images of H&E staining, and d) number of ED1‐positive inflammatory cells calculated from the sagittal images of immunohistochemistry. Scale bar = 1 mm. *p < 0.05 compared with control by Mann–Whitney U test.

**Figure 4:**
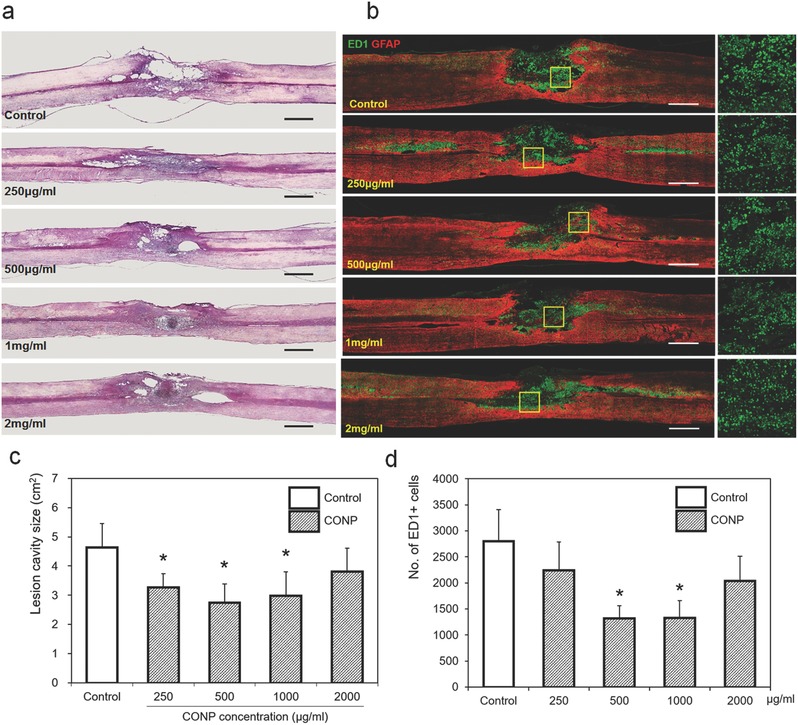
Representative a) H&E and b) immunohistochemical images of the injured spinal cord eight weeks after the application of CONPs with different concentrations from 250 to 2000 µg mL^−1^. The yellow boxes magnified on right side. c) Size of lesion cavity measured from the sagittal images of H&E staining, and d) number of ED1‐positive inflammatory cells calculated from the sagittal images of immunohistochemistry. Scale bar = 1 mm. *p < 0.05 compared with control by Mann–Whitney U test.

One week after the treatment, a large‐sized lesion cavity was formed in the control injury group; however, the CONP‐treated groups showed highly reduced lesion cavity (**Figure** **3**a). The measurement of lesion cavity size revealed substantial reduction by the treatment of CONPs at concentrations up to 1000 µg mL^−1^; however the cavity size increased with higher concentrations of CONPs (Figure 3c). Moreover, the immunostaining of cells positive for ED1 (in green), a marker for inflammatory cells, showed a large number of cells in the lesion cavity for the control group, which however, substantially decreased in the CONPs‐treated groups (Figure 3b). A quantification of ED1‐positive cells revealed a significantly decreased number after the treatment of CONPs up to 1000 µg mL^−1^, over which, however, the inflammatory cell number increased (Figure 3d). Based on these findings, the representative concentrations of CONPs (at 250, 500, 1000, and 2000 µg mL^−1^) were determined for the long term (eight weeks) in vivo experiment. At eight‐week postinjury, the treatment of CONPs at all the concentrations decreased the cavity size (**Figure** **4**a,c) and ED1‐positive cell number (Figure 4b,d); in particular, the intermediate concentrations of 500 and 1000 µg mL^−1^ CONPs were most effective, in both reducing cavity size and decreasing inflammatory cell number by ≈50%–60%.

**Figure 5:**
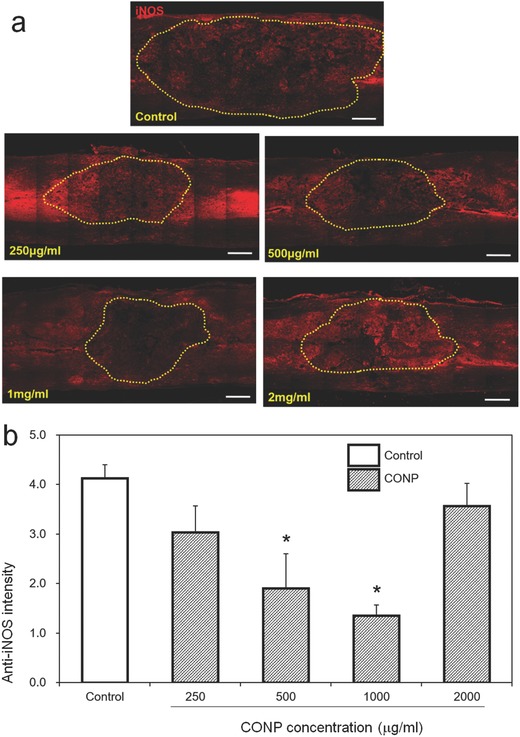
In vivo iNOS generation assay. a) Represent images of anti‐iNOS positive cells within injured spinal cord 24 h after the SCI. The lesion cavity outlined by yellow dots. b) Relative intensity of anti‐iNOS positive cells quantified. Scale bar = 500 µm. *p < 0.05 compared with control by Mann–Whitney U test.

To correlate the positive outcome at eight weeks with the acute phase of inflammation, we went back to measure the iNOS level at the injury site. At one day of SCI, a large number of cells were stained positive for iNOS in the injured control group; however, the stained cells decreased in the groups treated with CONPs (**Figure** **5**a). A quantification of the images revealed significant decrease in the iNOS expression after the treatment of CONPs up to 1000 µg mL^−1^, down to ≈30% of control group, but the higher concentration (2000 µg mL^−1^) was not effective, being comparable to the control (Figure 5b). The change in iNOS expression with respect to the CONP concentration was similarly observed with that in cavity size and inflammatory cell number. The findings confirmed the effective role of CONPs at moderate doses in suppressing the iNOS generation and subsequently reducing the cavity size and inflammatory cell population in the injured spinal cord.

### 2.5 Locomotor Functions of Injured Spinal Cord Are Significantly Improved by the CONPs

**Figure 6:**
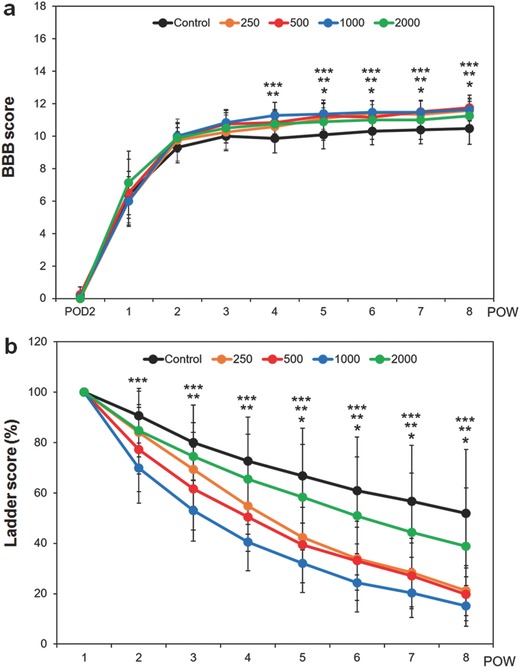
Locomotor functions of spinal cord injured rats after the application of CONPs until eight weeks. a) BBB score, and b) ladder score. *p < 0.05 between 250 µg mL^−1^ and control, **p < 0.05 between 500 µg mL^−1^ and control, and ***p < 0.05 between 1000 µg mL^−1^ and control, by Kruskal–Wallis test with Bonferroni correction.

The locomotor functions of the injured spinal cord with the treatment of CONPs were then examined up to eight weeks in terms of Basso, Beattie and Bresnahan (BBB) and ladder score. The CONP‐treated groups showed significantly higher BBB scores than the control at 500 and 1000 µg mL^−1^ from four weeks, and at 250 µg mL^−1^ from five weeks of postinjury (**Figure** **6**a). More noticeable effects were observed in the ladder score. The CONP‐treated groups showed significantly lower ladder scores than the control; from two weeks postinjury at 1000 µg mL^−1^, three weeks at 500 µg mL^−1^, and five weeks at 250 µg mL^−1^ (Figure 6b).

The treatment of CONPs to SCI has thus shown to elicit significant effects on the improvement of locomotor functions. The concentrations involved to be effective are also in line with those observed to enhance anti‐inflammation, suggesting the role of CONPs in improving the in vivo behavioral functions is closely related with the suppression of inflammatory reactions. Intriguing in the behavioral results is that the improvement in ladder score is rapid and noticeable, which means the coordination of weight‐supported hind limb is more improved in CONP‐treated SCI rats than in controls early from two weeks of postinjury. In our previous study, when induced neural stem cells were applied to in vivo rat spinal cord contusion models, the locomotor improvements were prominent from ten weeks of postinjury for the BBB score and from nine weeks for the ladder score.^18^ In fact, the inflammatory process following SCI is severe, and the infiltration of macrophages/microglia and T cells peaks at 7 and 9 d postinjury, respectively;^19^ further, scar formation, which initiates during this initial phase, may limit the stem cell‐derived regeneration process. Therefore, stem cell transplantation is often recommended ≈9–14 d after the injury;^20^ to overcome this, anti‐inflammatory approaches such as the treatment of CONPs may be beneficial at this acute stage, which can ultimately mitigate the stem cell survival conditions against inflammation.

In the acute injured central nervous system like SCI, the injury‐induced ROS, including nitric oxide, peroxynitrite, hydrogen peroxide, hydroxyl radical, and lipid peroxyl, are implicated to produce and aggravate many pathologic conditions, which leads to locomotor dysfunctions.^21^ For this reason, some antioxidative treatments involving the use of pharmacologic agents such as melatonin and curcumin have been tried in the animal SCI models to reduce ROS and concomitant functional deteriorations following SCI.^22^ In clinical settings, methylprednisolone (MP), a well‐known anti‐inflammatory agent during acute stage, also shows some antioxidative effects at higher doses;^23^ however, the high dose regimen is not recommended as a standard protocol now due to the adverse effects with relatively weak evidence of clinical improvements.^24^ To reduce the dose of MP, controlled release nanoparticles have also been applied to SCI models.^25^ One study reported that MP‐loaded nanoparticles enabled sustained release of MP, reduced the volume of lesion cavity, and improved locomotor function more than the single or systemic application of MP.^25^ Without the use of such therapeutic drugs, only with nanoparticles “CONPs,” we showed here for the first time the effective role in improving locomotor functions of injured spinal cord; therefore, as a further study, the CONPs can also be considered a promising candidate for controlled delivery of anti‐inflammatory drugs, to achieve more effective and synergistic functions.

### 2.6 CONPs in Injured Spinal Cord Hit the Molecular Pathway through Regulation of Proinflammatory Cytokines and ROS Level

**Figure 7:**
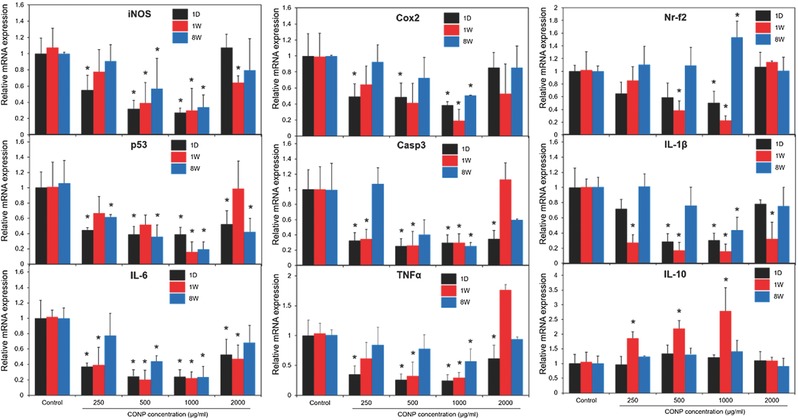
In vivo mRNA expression levels of genes associated with anti‐inflammatory and apoptosis, including ROS, Cox2, Nr‐f2, p53, Casp3, IL‐1β, IL‐6, TNFα, and IL‐10 within the injured spinal cord treated with CONPs at different concentrations (250–2000 µg mL^−1^), measured at one day, one week, and eight weeks postinjury. *p < 0.05 compared with control at the same period by Mann–Whitney U test.

We further analyzed the cellular phenomena involved in the ROS and inflammatory reactions in vivo, to better correlate with the behavioral outcome of improved locomotor functions. The expressions of a series of genes involved in ROS generation, apoptosis, inflammation, and regeneration, were analyzed at one day, one week, and eight weeks postinjury, using real‐time polymerase chain reaction (PCR) analysis (as profiled in **Figure** **7**).

Most genes including iNOS, cyclooxygenase‐2 (Cox2), nuclear factor (erythroid‐derived 2)‐like 2 (Nr‐f2), p53, caspase 3 (Casp3), interleukin 1 beta (IL‐1β), interleukin 6 (IL‐6), tumor necrosis factor alpha (TNF‐α), and leukemia inhibitory factor (LIF), except interleukin 10 (IL‐10), were downregulated in the CONP‐treated groups. The analyzed genes are known to get involved in some of key molecular and biological events in the injured spinal cord, including inflammation, apoptosis, and neuronal regeneration.^26^ The effects on gene regulation were conspicuous particularly at short periods (within one week), when the acute inflammatory related events were dominant in the injured site.^26^ Moreover, the up‐/downregulation of genes became more significant with increasing the concentration of CONP up to 1000 µg mL^−1^. However, at a higher concentration (2000 µg mL^−1^), the regulatory effect of CONP was not well demonstrated, presumably due to that the high‐dose related toxicity might complicate the anti‐inflammatory role of CONP.

Above all, the significant downregulation of mRNA level of iNOS through the CONP‐treatment, combined with the evidence of reduced iNOS‐producing cells in the immunocytochemistry (as provided in Figure 5), suggests the possible role of CONPs in scavenging ROS generated in vivo. This however needs clarification with the quantitative analysis of tissue ROS levels which warrants future studies.

Cox2, Nr‐f2, p53, and Casp3 are activated following SCI and are involved in the complex events of inflammatory, apoptosis and/or regeneration processes. More specifically, Cox2 is a cytokine for the synthesis of prostaglandins and increases right after SCI (starts to increase within 30 min, peaks at 3 h following SCI),^27^ Nr‐f2 modulates inflammation, apoptosis, and regeneration,^28^ p53 regulates microglia and macrophage proliferation and mitochondrial apoptosis,^29^ and Casp3 is an apoptotic cytokine.^30^ Furthermore, those activated molecules are heavily involved consequently in deteriorating the motor functions.[[qv: 29a]] A previous study revealed that secondary injury following SCI increased hydrogen peroxide and hydroxyl radicals adjacent to the lesion site, leading to apoptosis and necrosis of neurons and astrocytes.[[qv: 4a]] Therefore, the downregulated apoptotic genes might result from the reduced ROS levels by the CONPs.

Likewise, the proinflammatory cytokines including IL‐1β, IL‐6, TNF‐α, and LIF, those known to dramatically increase after SCI, were substantially reduced by the CONPs. IL‐6 is one of the proinflammatory cytokines that can increase lesion volume and interfere with axonal regrowth following SCI,^31^ and TNF‐α, usually elevated within several hours following SCI, is critically involved in apoptosis in neurodegenerative diseases. On the contrary, IL‐10 is an anti‐inflammatory cytokine and promotes neuronal survival following SCI.^32^ Therefore, the current observation that CONPs downregulate molecules involved in ROS generation and apoptotic/proinflammation, and simultaneously, upregulate anti‐inflammatory cytokine, reflects well the regulatory roles of CONPs played in those events and the possible consequence in the locomotor functions.

Another aspect in the SCI is the polarization of macrophages, i.e., from M1 to M2 phase, which is also possible in the current CONP‐involved events. The number of M1 macrophages peaks at 5 d, promoting inflammation by activating proinflammatory cytokines such as IL‐1β, IL‐6, and TNF‐α, and apoptosis by ROS production; whereas, the number of M2 macrophages peaks at 14 d, substantially producing IL‐10 to promote axonal regeneration.^33^ Thus, the directly injected CONPs, when internalized to macrophages, can activate their phenotype more anti‐inflammatory (i.e., more M2 than M1), ultimately promoting axonal regeneration. This issue is of interest and value to elucidate the therapeutic mechanism of CONPs in the complicate molecular events during SCI repair, and needs further exploration.

**Figure 8:**
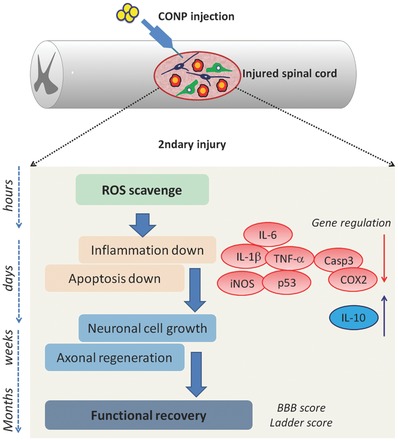
Schematic illustration showing the series of events involved in secondary injury after spinal cord contusion and the therapeutic regulation of directly injected CONPs through ROS scavenging, suppressing inflammation and apoptosis, enhancing neuronal cell growth and axonal regeneration, and consequently functional recovery.

The roles of CONPs in the series of events within SCI, and the possible molecular actions involved, as deduced from the findings demonstrated above, are illustrated in **Figure** **8**. The conventional approach of injecting anti‐inflammatory drug to the injury site is fraught with adverse effects and thus not recommended now. Therefore, the therapeutic effects of CONPs evidenced in the current study would warrant further preclinical investigations involving larger animal models. Moreover, it would be fruitful to clarify the synergistic or additive role of CONPs with clinically‐available drugs in further improving the treatment for SCI.

## 3 Conclusions

It is thus summarized that CONPs administered to a contused spinal cord of rats could significantly improve the locomotor functions in terms of BBB and ladder score up to eight weeks. The expression of acute inflammatory and apoptotic regulatory molecules (Cox2, Nr‐f2, P53, Casp3, IL‐1β, IL‐6, and TNF‐α) were substantially suppressed in vivo particularly at the early stage of SCI by the CONP‐treatment, which in turn led to the reduction of contusion lesion size and inflammatory cells. These therapeutic effects are attributed to the critical role of CONPs in scavenging ROS, as evidenced by the downregulation of iNOS at the gene and protein levels in vivo. There was an optimal therapeutic dose range for this improvement (500–1000 µg mL^−1^), above which the CONPs adversely affected the functional recovery. Supporting the in vivo findings was the in vitro reduction in iNOS expression and increase in neuronal survival. As a simple approach with translational potential, local administration of CONPs at a proper dose may augment any drug, gene, or cell therapy for acute SCI.

## 4 Experimental Section

*Synthesis and Characterizations of CONPs*: CONPs were synthesized using the hydrothermal process.^34^ In brief, cerium nitrate was dissolved in distilled water at 1 × 10^−3^ m, with pH adjusted at 8.0 using ammonium hydroxide. Separately, CTAB was dissolved in distilled water at 0.1 × 10^−3^ m. Two solutions were mixed dropwise in a Teflon hydrothermal vessel with a total volume of 60 mL. The Teflon vessel was transferred to a stainless steel autoclave and thermal‐treated at 140 °C for 24 h under autogenous pressure conditions to obtain hydrothermal‐processed CONPs. The nanoparticle morphology was examined by high‐resolution TEM (JEOL 7100, Jeol Ltd., Tokyo, Japan) after dispersing in anhydrous ethanol and then dropped on a copper grid. The size of nanoparticles was measured from TEM images (*n* = 30). The selected area electron diffraction pattern of the crystals was also analyzed. The crystal structure of nanoparticles was determined by X‐ray powder diffraction (Ragaku Co. Ltd., Tokyo, Japan). XPS (ESCA 2000, Thermo Fisher Scientific, Waltham, MA, USA) was carried out to analyze the chemical ionic status of Ce. The surface electrical properties of the nanoparticles were observed by means of ζ‐potential measurement (Zetasizer Nano, Malvern Instruments Ltd., Malvern, UK) at pH 7.0 and 25 °C. The hydrodynamic size of the nanoparticles was also characterized using the Zetasizer Nano by a DLS method. Nanoparticles of 100 µg dispersed in DW or in neurobasal medium were measured at 25 °C (*n* = 4). The enzyme mimetic activity of CONPs was evaluated by monitoring the redox reaction between TMB and H_2_O_2_ in the presence of CONPs.^35^ The reaction was first monitored time‐dependently (every 5 min up to 20 cycles) using UV–vis spectroscopy (Varian Cary 100, Varian Analytical Instruments, Walnut Creek, CA, USA) at a broad wavelength scan. A typical solution was made of 1 mL acetate buffer solution (50 × 10^−3^ m, pH = 4.0), 25 µg nanoparticles, 0.5 × 10^−6^ m TMB, and 1 mol H_2_O_2_,^36^ which was used when filtered through a syringe filter (pore size 0.45 µm; Hyundai Micro Co., Ltd., Seoul, Korea). Based on this, the reaction was again monitored at a wavelength of 652 nm with varying H_2_O_2_ concentrations (10 × 10^−6^, 20 × 10^−6^, 40 × 10^−6^, 60 × 10^−6^, 80 × 10^−6^, 100 × 10^−6^, 200 × 10^−6^, 400 × 10^−6^, 800 × 10^−6^, and 1000 × 10^−6^ m) using UV–vis spectroscopy (Biochrom, Libra S22, Cambridge, UK).

*Primary Cultures of Rat Cerebral Cortical Neurons*: Cortical neurons were isolated and cultured using a modified method from the previous study.^37^ Cortex was removed from the Sprague‐Dawley rat embryos (embryonic day 16) and placed into Hank's balanced salt solution (HBSS) (GIBCO, Grand Island, NY, USA), and meninges were manually removed. Cortex was rinsed twice in HBSS medium and then transferred to a 15 mL conical tube containing 2 mL of 2.5 mg mL^−1^ (in HBSS) papain solution (Sigma‐Aldrich, St. Louis, MO, USA). After incubation for 15 min at 37 °C, the papain solution was discarded and the remaining samples were rinsed twice in 2 mL HBSS, and then centrifuged at 1500 rpm for 3 min to discard HBSS. The samples were placed in 1 mL cortical neuron culture media containing Neurobasal medium (Gibco, Waltham, MA, USA) supplemented with B27 (Invitrogen Life Technologies, Carlsbad, CA, USA), Gluta‐MAX (Invitrogen Life Technologies), and 1% penicillin/streptomycin. The pellet was resuspended by triturating about 20 times through 1 mL pipette tips. The single cells were then plated onto 18 mm circular cover slips for immune staining and onto 96‐well plates for cell viability assay. The cover slips and plates were prepared by coating with 20 mg mL^−1^ poly‐d‐lysine (Sigma‐Aldrich) overnight and 10 mg mL^−1^ laminin (Sigma‐Aldrich) for 2 h at 4 °C.

*Hydrogen Peroxide (H_2_O_2_)‐Induced Neuronal Injury and CONP Treatments*: Cortical neurons were cultured in 96‐well plates at a density of 5 × 104 cells per well in the cortical culture medium for 2 h. Then the cortical neuron medium was replaced with H_2_O_2_ that was prepared at different concentrations in cortical neuron culture medium (100 × 10^−6^, 250 × 10^−6^, and 500 × 10^−6^ m) for 30 min or 1 h. After this, the cortical neuron culture medium was replaced with various concentrations of CONPs (1, 10, 25, 50, 100, 250, 500, 1000, 2000, and 4000 µg mL^−1^ for MTT assay, and 10, 250, and 1000 µg mL^−1^ for live/dead cell assay) and then incubated in 5% CO_2_ at 37 °C for 24 h. To perform MTT assay, MTT was dissolved in cortical neuron culture medium and added at final concentration of 0.5 mg mL^−1^ at 37 °C for 4 h. Afterward, the MTT contained cortical neuron culture medium was replaced by 100 µL dimethyl sulfoxide (Sigma‐Aldrich). Optical density was measured at 570 nm by a Universal Microplate Reader. For live and dead cell assay, a fluorescent live/dead cell assay kit (L3224, Invitrogen Life Technologies) was used. Treated cells were incubated with 2 × 10^−6^ m Calcein‐AM and 4 × 10^−6^ m EthD‐1 in Dulbecco's phosphate‐buffered saline (DPBS) for 20 min at room temperature, and the prepared samples were visualized under a confocal microscope (Carl Zeiss Inc., Oberkochen, Germany) at 488 nm excitation (green) and 555 nm (red) wavelengths. Three images were taken from each well (three wells for each condition), and the number of cells labeled with green (live) or red (dead) color was counted using an ImageJ software (1.37 v, National Institutes of Health, Bethesda, MD, USA).

*iNOS Immunostaining and Analysis*: After the treatment of 500 × 10^−6^ m H_2_O_2_ solution for 30 min, the CONPs were administered at varying concentrations (1, 10, 25, 50, 100, 250, and 500 µg mL^−1^) and then incubated in 5% CO_2_ at 37 °C. After 12 h, the cortical neuron samples were fixed with 4% paraformaldehyde for 30 min, and then rinsed three times for 5 min each with phosphate‐buffered saline (PBS)g. The cells were permeabilized in 0.2% Triton X‐100 (dissolved in 2% normal goat serum/PBS solution) for 5 min, washed three times in PBS for 5 min, and blocked in 2% normal goat serum/PBS solution for 1 h. Primary antibodies (mouse anti‐SMI312, 1:1000, Covance, Princeton, NJ, USA; rabbit anti‐iNOS, 1:100, Abcam, Cambridge, MA, USA) diluted in 2% normal goat serum/PBS solution were incubated overnight at 4 °C and washed three times in PBS. A secondary antibody (FITC‐conjugated goat anti‐mouse IgG, 1:200; Rhodamine Red‐X‐conjugated affinipure goat anti‐rabbit IgG, 1:200, Jackson Immuno‐Research Labs, Inc., West Grove, PA, USA) diluted in 2% normal goat serum/PBS solution was incubated at room temperature for 2 h, then washed three times with PBS. The coverslips were treated with 4′‐6‐diamidino‐2‐phenylindole containing PBS at room temperature for 10 min, washed three times with PBS, covered with fluorescent mounting medium (Dako Cytomation, Carpinteria, CA, USA), and then observed under a confocal microscopy (Carl Zeiss Inc.). For the quantification of iNOS fluorescence intensity, four randomized images at each group were captured with 400× magnification and the average intensity of iNOS fluorescence was measured using ImageJ software (National Institutes of Health).

*In Vivo Models of Spinal Cord Contusion and Local Delivery of CONPs*: Adult female Sprague‐Dawley rats (12‐week old, 230–250 g) were used in all experiment. All procedures complied with Dankook University's Institutional Animal Care and Use Committee (Approval No. DKU‐14‐035). Animals were housed individually in a temperature‐controlled environment (23–25 °C) and humidity (45%–50%) under 12 h light/dark cycle with ad libitum water and food access.

Surgical procedures have been previously described in detail.^38^ Briefly, rats were deeply anesthetized by isoflurane (Forane; Choongwae Pharma, Seoul, Korea) inhalation and laminectomy was performed at T9–T10 level. All animals received a moderate contusion injury (200 kdyn) to expose T9 spinal cord using the Infinite Horizon impactor (IH‐400, Precision Systems and Instrumentation, LLC, KY, USA). CONPs with different concentrations (50, 100, 250, 500, 1000, 2000, and 4000 µg mL^−1^) were prepared in distilled water immediately before use. At 30 min following contusion, a total volume of 10 µL CONPs solution directly injected into the lesion cavity at T9 spinal cord (subdural, and exactly intralesional) via Hamilton syringe at a rate of 1 µL min^−1^ (Hamilton Company, Reno, NV, USA). Control animals received the same amount of distilled water without CONPs. After delivering the solution, the cord was then covered with a piece of hemostatic agent (Surgicel, Johnson and Johnson, Arlington, TX, USA), and the muscle and subcutaneous layers, skin were closed by layer. Intramuscular injection of 40 mg kg^−1^ cefotiam hydrochloride (Fontiam, Hanmi Pharma, Seoul, Korea) was performed to all operated rats for 3 d and intraperitoneal injection of normal saline (3 mL) was made just after surgery. Animals also received oral administration of 10 mg kg^−1^ acetaminophen syrup (Tylenol, Janssen Pharmaceutica, Titusville, NJ, USA) for 3 d, and bladder expression was performed twice a day and continued until the amount of expressed urine was less than 0.5 mL per day. These groups of rats were sacrificed at one week (*n* = 9 per group). Based on the results on one week, additional SCI models were made for one day (*n* = 8 per group) and for eight weeks (*n* = 9 per group), with the optimal concentrations of CONPs.

*Histology and Immunofluorescence*: Frozen sections were used for hematoxylin and eosin staining (H&E) and immunohistochemistry. Five rats in each group were perfused with 0.9% saline followed by 4% paraformaldehyde (Hushi Inc., Shanghai, China) in 0.1 m PBS (pH 7.4). The spinal cord was then dissected, post‐fixed overnight in 4% paraformaldehyde at 4 °C, and transferred to 30% sucrose in 0.1 m PB for 3 d. The cord was embedded M1 compound (Thermo Fisher Scientific Inc.) and cryosectioned into 16 µm in the sagittal plane. H&E stain was performed to examine the lesion site of the injured spinal cord at one week and eight weeks postinjury. The sections were stained with hematoxylin for 5 min, rinsed in running tap water for 3 min, and then stained with eosin for 1 min. The stained sections were dehydrated through a graded series of ethanol, cleared with xylene, and then imaged under a microscope (Nikon, Tokyo, Japan). The lesion cavity in H&E‐stained sections (*n* = 3 per group) was outlined manually under a light microscope at X100 magnification, and the area was calculated using the National Institutes of Health ImageJ software (National Institutes of Health), as described elsewhere.^39^

Immunohistochemistry was used to analyze the inflammatory response in contused spinal cord. The primary antibodies, rabbit anti‐iNOS (1:100, Abcam), rabbit anti‐GFAP (1:1000, Dako), mouse anti‐ED1 (1:400, Merck Millipore, Temecula, CA, USA), were incubated overnight at 4 °C. After the sections were washed three times, goat anti‐rabbit (Alexa Fluor 546) and goat anti‐mouse (Alexa Fluor 488) secondary antibodies were used at a dilution of 1:200 in 2% normal gout serum in PBS. Following 2 h incubation, the sections were washed three times with PBS. Stained tissue sections were imaged using a confocal microscopy (Carl Zeiss Inc.). For quantitation of ED1+ monocytes and macrophages in the sagittal section, the images were captured at the lesion site using 100× magnification on a confocal microscope, and then counting the expressed cell numbers (per 1 mm^2^) manually. For quantification of iNOS fluorescence intensity in the sagittal section, three representative images from the lesion site per animal (*n* = 3 per group) were captured with 400× magnification and fixed acquisition settings using confocal microscope and iNOS fluorescence intensity was analyzed using ImageJ software (National Institutes of Health). The background subtraction was performed using a rolling ball algorithm of ImageJ tools and the average intensity was measured with ImageJ measurement.

*Assessments of Locomotor Functions*: For the evaluation of locomotor functions of paralyzed hindlimb after spinal cord injury, two scales were used: Basso, Beattie, and Bresnahan (BBB) scale and horizontal ladder test. The BBB scale of no hindlimb movement is 0, and that of normal hindlimb movement is 21.^40^ Rats were analyzed by two observers who were blinded to the treatment received by each rat and positioned across from each other to observe both sides of the rats during 4 min walking in the open field (cylindrical‐shaped acrylic box; 90 cm diameter, 15 cm high) with a smooth floor. Horizontal ladder test was performed on a runway made of acryl walls (10 cm tall, 127 cm long, 8 cm wide between walls, 1 cm between rungs).^41^ All rats were trained to walk from left to right on a runway several times for adaptation before testing and then captured with a digital camcorder. The ladder score was calculated as below

(1) $$\mathrm{Ladder}\;\;\mathrm{score}=\mathrm{Erroneous}\;\;\mathrm{steps}\;\;\mathrm{of}\;\;\mathrm{hind}\;\;\mathrm{limb}\;/\;\mathrm{total}\;\;\mathrm{steps}\;\;\mathrm{of}\;\;\mathrm{hind}\;\;\mathrm{limb}\times \mathrm{100}\;\left(\%\right)$$

The locomotor function of each group was examined every 7 d until sacrifice. All locomotor tests were recorded for at least 4 min with a digital camcorder for coupling score and ladder score and were interpreted by two observers who were blinded to the identity of the rats.

*RNA Isolation and Real‐Time PCR*: To examine the effects of CONPs on the reactive oxygen species (ROS), apoptosis and inflammation in SCI rat models, the expression level of nine genes; iNOS, Cox2, Nr‐f2, p53, Casp3, IL‐1β, IL‐6, IL‐10, and TNF‐α were evaluated in spinal cord tissues using real‐time PCR (**Table** **1**). Briefly, total RNA was extracted from spinal cord by using an RNeasy mini kit (Qiagen, Hilden, Germany). cDNA was synthesized using random hexamer primers and SuperScript III (Invitrogen, Thermo Fisher Scientific). All primers pairs were designed using the UCSC Genome Bioinformatics and the NCBI database. Real‐time PCR was performed using Fast SYBR Green Master Mix (Applied Biosystems, Thermo Fisher Scientific) on a StepOne Real‐Time PCR system (Thermo Fisher Scientific). Each real‐time PCR was performed on at least triplicate assay (*n* = 3 for each group). The expression of each target gene was normalized to glyceraldehyde 3‐phosphate dehydrogenase (GAPDH) and expressed as the fold change relative to the control groups.

**Table 1:** Primer sequences used for real time PCR of tissue samples

Gene	5′‐3′	Primer sequence
iNOS	Forward	CTCAGCACAGAGGGCTCAAAG
	Reverse	TGCACCCAAACACCAAGGT
Cox2	Forward	GGCCATGGAGTGGACTTAAA
	Reverse	CTCTCCACCGATGACCTGAT
Nr‐f2	Forward	TCCAGACAGACACCAGTGGA
	Reverse	GGAATGTCTCTGCCAAAAGC
p53	Forward	ACAGCGTGGTGGTACCGTAT
	Reverse	GGAGCTGTTGCACATGTACT
Casp 3	Forward	GAACGCGAAGAAAAGTGACC
	Reverse	GAGTCCATCGACTTGCTTCC
IL‐1β	Forward	GCCCGTCCTCTGTGACTCGT
	Reverse	TGTCGTTGCTTGTCTCTCCTTGTA
IL‐6	Forward	ACCACCCACAACAGACCAGT
	Reverse	CAGAATTGCCATTGCACAAC
IL‐10	Forward	CAGCTGCGACGCTGTCATCG
	Reverse	GCAGTCCAGTAGATGCCGGGT
TNF‐α	Forward	CTCAAGCCCTGGTATGAGCC
	Reverse	GGCTGGGTAGAGAACGGATG
GAPDH	Forward	CACTGAGCATCTCCCTCACA
	Reverse	GAGGGTGCAGCGAACTTTAT

*Statistical Analyses*: All numeric data were reported as means ± SDs, and IBM SPSS Statistics 21 (International Business Machines Corp., Armonk, NY, USA) was used for the analysis. The Shapiro‐Wilk test was performed to check normal distribution of all quantified histological and functional data from each group, and according to the result, parametric or nonparametric tests were chosen. For histological, immunohistochemical and quantitative PCR data, Mann–Whitney U test was used to detect the differences between control and CONPs‐treated experimental groups. To compare the anti‐iNOS intensity and the relative cell viability of CONPs‐treated groups with untreated and H_2_O_2_‐treated controls, the Kruskal–Wallis test with Bonferroni correction method was used. The repeated measures one‐way analysis of variance were used to compare locomotor functions including the BBB and the ladder score tests among the control and experimental groups, and then the Kruskal–Wallis test with Bonferroni correction method was used at each time point. Significance was determined at *p* < 0.05.

## Conflict of Interest

The authors declare no conflict of interest.

## Supporting information

Supplementary

## References

[1] C. H. Ho , L. A. Wuermser , M. M. Priebe , A. E. Chiodo , W. M. Scelza , S. C. Kirshblum , Arch. Phys. Med. Rehabil. 2007, 88, S49. 17321849 10.1016/j.apmr.2006.12.001

[2] N. A. Silva , N. Sousa , R. L. Reis , A. J. Salgado , Prog. Neurobiol. 2014, 114, 25. 24269804 10.1016/j.pneurobio.2013.11.002

[3] J. M. Schwab , Y. Zhang , M. A. Kopp , B. Brommer , P. G. Popovich , Exp. Neurol. 2014, 258, 121. 25017893 10.1016/j.expneurol.2014.04.023 PMC4099970

[4] a) F. Bao , D. Liu , Neuroscience 2004, 126, 285; 15207346 10.1016/j.neuroscience.2004.03.054

[5] a) D. Liu , F. Bao , Neuroscience 2015, 285, 81; 25451281 10.1016/j.neuroscience.2014.10.063 PMC4304797

[6] a) G. Ciofani , G. G. Genchi , I. Liakos , V. Cappello , M. Gemmi , A. Athanassiou , B. Mazzolai , V. Mattoli , Pharm. Res. 2013, 30, 2133; 23661146 10.1007/s11095-013-1071-y

[7] K. L. Heckman , W. DeCoteau , A. Estevez , K. J. Reed , W. Costanzo , D. Sanford , J. C. Leiter , J. Clauss , K. Knapp , C. Gomez , P. Mullen , E. Rathbun , K. Prime , J. Marini , J. Patchefsky , A. S. Patchefsky , R. K. Hailstone , J. S. Erlichman , ACS Nano 2013, 7, 10582. 24266731 10.1021/nn403743b

[8] C. K. Kim , T. Kim , I. Y. Choi , M. Soh , D. Kim , Y. J. Kim , H. Jang , H. S. Yang , J. Y. Kim , H. K. Park , S. P. Park , S. Park , T. Yu , B. W. Yoon , S. H. Lee , T. Hyeon , Angew. Chem., Int. Ed. 2012, 51, 11039. 10.1002/anie.201203780 22968916

[9] F. Pagliari , C. Mandoli , G. Forte , E. Magnani , S. Pagliari , G. Nardone , S. Licoccia , M. Minieri , P. Di Nardo , E. Traversa , ACS Nano 2012, 6, 3767. 22524692 10.1021/nn2048069

[10] a) S. M. Hirst , A. S. Karakoti , R. D. Tyler , N. Sriranganathan , S. Seal , C. M. Reilly , Small 2009, 5, 2848; 19802857 10.1002/smll.200901048

[11] a) E. J. Park , J. Choi , Y. K. Park , K. Park , Toxicology 2008, 245, 90; 18243471 10.1016/j.tox.2007.12.022

[12] a) S. S. Lee , W. Song , M. Cho , H. L. Puppala , P. Nguyen , H. Zhu , L. Segatori , V. L. Colvin , ACS Nano 2013, 7, 9693; 24079896 10.1021/nn4026806

[13] I. Celardo , J. Z. Pedersen , E. Traversa , L. Ghibelli , Nanoscale 2011, 3, 1411. 21369578 10.1039/c0nr00875c

[14] S. Singh , A. Kumar , A. Karakoti , S. Seal , W. T. Self , Mol. BioSyst. 2010, 6, 1813. 20697616 10.1039/c0mb00014k PMC3039285

[15] M. Leirós , E. Alonso , J. A. Sanchez , M. E. Rateb , R. Ebel , W. E. Houssen , M. Jaspars , A. Alfonso , L. M. Botana , ACS Chem. Neurosci. 2014, 5, 71. 25387680 10.1021/cn500258c

[16] a) M. Horie , K. Nishio , H. Kato , K. Fujita , S. Endoh , A. Nakamura , A. Miyauchi , S. Kinugasa , K. Yamamoto , E. Niki , Y. Yoshida , Y. Hagihara , H. Iwahashi , J. Biochem. 2011, 150, 461; 21693544 10.1093/jb/mvr081

[17] G. Cheng , W. Guo , L. Han , E. Chen , L. Kong , L. Wang , W. Ai , N. Song , H. Li , H. Chen , Toxicol. In Vitro 2013, 27, 1082. 23416263 10.1016/j.tiv.2013.02.005

[18] J. Y. Hong , S. H. Lee , S. C. Lee , J. W. Kim , K. P. Kim , S. M. Kim , N. Tapia , K. T. Lim , J. Kim , H. S. Ahn , K. Ko , C. Y. Shin , H. T. Lee , H. R. Scholer , J. K. Hyun , D. W. Han , J. Biol. Chem. 2014, 289, 32512. 25294882 10.1074/jbc.M114.588871 PMC4239606

[19] K. D. Beck , H. X. Nguyen , M. D. Galvan , D. L. Salazar , T. M. Woodruff , A. J. Anderson , Brain 2010, 133, 433. 20085927 10.1093/brain/awp322 PMC2858013

[20] M. Nakamura , H. Okano , Cell Res. 2013, 23, 70. 23229514 10.1038/cr.2012.171 PMC3541652

[21] M. Bains , E. D. Hall , Biochim. Biophys. Acta 2012, 1822, 675. 22080976 10.1016/j.bbadis.2011.10.017 PMC4134010

[22] a) B. Cemil , K. Topuz , M. N. Demircan , G. Kurt , K. Tun , M. Kutlay , O. Ipcioglu , Z. Kucukodaci , Acta Neurochir. 2010, 152, 1583; 20535508 10.1007/s00701-010-0702-x

[23] E. Kaptanoglu , M. Tuncel , S. Palaoglu , A. Konan , E. Demirpence , K. Kilinc , J. Neurosurg. 2000, 93, 77. 10.3171/spi.2000.93.1.0077 10879762

[24] M. Bydon , J. Lin , M. Macki , Z. L. Gokaslan , A. Bydon , World Neurosurg. 2014, 82, 848. 23454689 10.1016/j.wneu.2013.02.062

[25] a) Y. T. Kim , J. M. Caldwell , R. V. Bellamkonda , Biomaterials 2009, 30, 2582; 19185913 10.1016/j.biomaterials.2008.12.077 PMC2678685

[26] O. N. Hausmann , Spinal Cord 2003, 41, 369. 12815368 10.1038/sj.sc.3101483

[27] K. Adachi , Y. Yimin , K. Satake , Y. Matsuyama , N. Ishiguro , M. Sawada , Y. Hirata , K. Kiuchi , Neurosci. Res. 2005, 51, 73. 15596243 10.1016/j.neures.2004.10.007

[28] a) Y. Pomeshchik , I. Kidin , E. Savchenko , T. Rolova , M. Yamamoto , A. L. Levonen , S. Yla‐Herttuala , T. Malm , K. Kanninen , J. Koistinaho , Antioxid. Redox Signaling 2014, 20, 1313; 10.1089/ars.2013.5453 23841575

[29] a) E. M. Floriddia , K. I. Rathore , A. Tedeschi , G. Quadrato , A. Wuttke , J. M. Lueckmann , K. A. Kigerl , P. G. Popovich , S. Di Giovanni , J. Neurosci. 2012, 32, 13956; 23035104 10.1523/JNEUROSCI.1925-12.2012 PMC6704782

[30] L. Stefanis , Neuroscientist 2005, 11, 50. 15632278 10.1177/1073858404271087

[31] D. Bastien , S. Lacroix , Exp. Neurol. 2014, 258, 62. 25017888 10.1016/j.expneurol.2014.04.006

[32] a) C. D. Thompson , J. C. Zurko , B. F. Hanna , D. J. Hellenbrand , A. Hanna , J. Neurotrauma 2013, 30, 1311; 23731227 10.1089/neu.2012.2651

[33] Y. J. Chen , H. Zhu , N. Zhang , L. Shen , R. Wang , J. S. Zhou , J. G. Hu , H. Z. Lu , J. Neurosci. Res. 2015, 93, 1526. 26096572 10.1002/jnr.23612

[34] a) L. Yan , R. Yu , J. Chen , X. Xing , Cryst. Growth Des. 2008, 8, 1474;

[35] Z. Tian , J. Li , Z. Zhang , W. Gao , X. Zhou , Y. Qu , Biomaterials 2015, 59, 116. 25968461 10.1016/j.biomaterials.2015.04.039

[36] Y. Yang , Z. Mao , W. Huang , L. Liu , J. Li , J. Li , Q. Wu , Sci. Rep. 2016, 6, 35344. 27748403 10.1038/srep35344 PMC5066218

[37] S. Y. Xu , Y. M. Wu , Z. Ji , X. Y. Gao , S. Y. Pan , J. Biomed. Biotechnol. 2012, 2012, 803930. 23193366 10.1155/2012/803930 PMC3486623

[38] W. B. Park , S. Y. Kim , S. H. Lee , H. W. Kim , J. S. Park , J. K. Hyun , BMC Neurosci. 2010, 11, 119. 20846445 10.1186/1471-2202-11-119 PMC2955046

[39] J. Y. Hong , S. H. Lee , S. C. Lee , J.‐W. Kim , K.‐P. Kim , S. M. Kim , N. Tapia , K. T. Lim , J. Kim , H.‐S. Ahn , K. Ko , C. Y. Shin , H. T. Lee , H. R. Schöler , J. K. Hyun , D. W. Han , J. Biol. Chem. 2014, 289, 32512. 25294882 10.1074/jbc.M114.588871 PMC4239606

[40] D. M. Basso , M. S. Beattie , J. C. Bresnahan , Exp. Neurol. 1996, 139, 244. 8654527 10.1006/exnr.1996.0098

[41] K. Fouad , I. Klusman , M. E. Schwab , Eur. J. Neurosci. 2004, 20, 2479. 15525289 10.1111/j.1460-9568.2004.03716.x

