# Supplementary material for: Functional Recovery of Contused Spinal Cord in Rat with the Injection of Optimal‐Dosed Cerium Oxide Nanoparticles
Source: Adv Sci (Weinh). 2017 Jul 8;4(10):1700034. doi: 10.1002/advs.201700034 (PMC5644223; doi:10.1002/advs.201700034)
Supplement: Supplementary file 1 — Supplementary [file ADVS-4-na-s001.pdf]

Copyright WILEY-VCH Verlag GmbH & Co. KGaA, 69469 Weinheim, Germany, 2017.

ADVANCED SCIENCE
Open Access

Supporting Information

for *Adv. Sci.,* DOI: 10.1002/advs.201700034

Functional Recovery of Contused Spinal Cord in Rat with the Injection of Optimal-Dosed Cerium Oxide Nanoparticles

Jong-Wan Kim, Chinmaya Mahapatra, Jin-Young Hong, Min Soo Kim, Kam W. Leong, Hae-Won Kim, and Jung Keun Hyun**

Supplementary Data

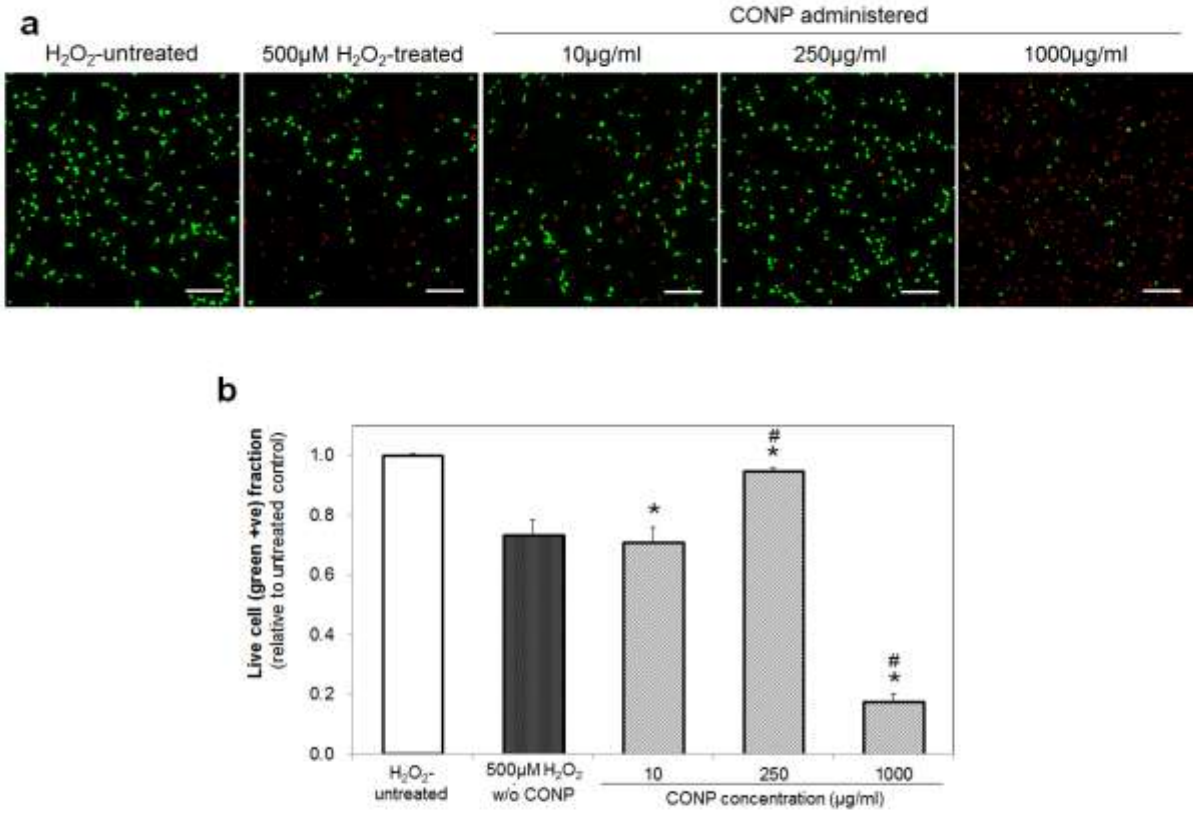

Figure S1. Live/dead cell assay of the 500 μM H_2O_2-treated cortical neurons with or without the CONP administration at various concentrations (10, 250, and 1,000 μg/ml) *in vitro*: (a) Representative images of live (green) and dead (red) cortical neurons (Scale bar = 100 μm), and (b) the relative live cell fractions (green-positive) compared (data presented w.r.t. H_2O_2-untreated control). $^*p < 0.05$ *vs.* untreated control group, and $^{\#}p < 0.05$ *vs.* H_2O_2-treated group, by Kruskal-Wallis test with Bonferroni correction.
